# The Diagnostic Role of the Pronator Quadratus Sign in Paediatric Wrist Fractures: A Retrospective Study

**DOI:** 10.3390/diagnostics16101530

**Published:** 2026-05-18

**Authors:** Viola Sbampato, Victor Starkenmann-Darbellay, Andreas Tsoupras, Wassim Ben Abdennebi, Anne Tabard-Fougère, Dimitri Ceroni, Christina Steiger

**Affiliations:** 1 Pediatric Orthopedic and Traumatology Unit, Geneva University Hospitals, University of Geneva, 1205 Geneva, Switzerland; 2 Department of Orthopedics and Traumatology, Valais Hospital, 1920 Martigny, Switzerland

**Keywords:** distal radius fracture, paediatric orthopaedics, fracture diagnostics

## Abstract

**Background/Objectives**: Wrist fractures are common paediatric injuries, yet some remain radiographically occult. The Pronator Quadratus Sign (PQS), defined by displacement or alteration of the fat stripe overlying the pronator quadratus muscle (PQm), remains a debated radiologic diagnostic marker. This study evaluated whether PQS can serve as a diagnostic adjunct for wrist fractures and whether specific PQS morphological types are associated with different fracture patterns. **Methods**: This retrospective study included paediatric patients presenting with wrist trauma. Radiographs were categorized into three groups: no fracture, torus fracture, and other non-displaced distal radius fractures. PQS morphology was classified using a six-tier system. Quantitative assessment included measurement of the anterior distance from the distal radius to the external border of the pronator quadratus muscle (X) and radial width (R), expressed as the X/R ratio to standardize comparisons. **Results**: A total of 247 patients were included. The X/R ratio differed significantly among groups (*p* < 0.001), with higher values observed in patients with fractures. ROC analysis demonstrated good discrimination performance between no fracture and non-displaced distal radius fractures (AUC = 0.80), but limited performance for torus fractures (AUC = 0.62). Although PQS morphology alone was not a reliable indicator of fracture presence, the distribution of PQS types—representing distinct patterns of pronator quadratus fat stripe appearance and displacement—differed significantly across fracture groups (*p* < 0.001). **Conclusions**: Quantitative PQS assessment using the X/R ratio is associated with improved discrimination for non-displaced distal radius fractures, while PQS morphology alone shows limited diagnostic utility. Further prospective studies incorporating magnetic resonance imaging are warranted to clarify its role in occult fracture detection.

## 1. Introduction

Distal radius fractures are among the most common skeletal injuries in the paediatric population, accounting for approximately 25–40% of all fractures in children [1,2]. These injuries frequently result from falls onto an outstretched hand and represent a significant proportion of emergency department visits, requiring accurate diagnosis and appropriate management [3,4,5]. While standard radiographic evaluation using anteroposterior (AP) and lateral (Lat) X-rays remains the first-line diagnostic tool, some fractures remain occult in the early stages, prompting the need for indirect markers and further imaging [6,7,8,9].

The Pronator Quadratus Sign (PQS) has been described as an indirect radiographic indicator of distal radius and ulna fractures [5,6,10,11]. It is thought to result from the accumulation of fluid, such as oedema or haemorrhage, within the pronator quadratus muscle (PQm) following trauma [5]. This muscular thickening displaces the thin fat stripe normally located between the PQm and the overlying flexor digitorum profundus tendon [12], producing a radiolucent radiological sign that may precede radiographically visible fractures and serve as a potential early indicator of occult injury [5,6,10,13,14].

Since its initial description, the diagnostic value of the PQS has been widely investigated, with heterogeneous findings reported across studies [5,6,11,12,13]. Early reports suggested that the PQS may assist in detecting subtle or occult distal forearm injuries [5,13,14], whereas subsequent work has raised concerns regarding its reliability and diagnostic performance [11,15,16]. A recent systematic review and meta-analysis further highlighted its heterogeneous diagnostic performance, reporting moderate pooled specificity but variable sensitivity across studies and supporting its role as an adjunctive rather than standalone diagnostic marker for distal radius fractures [17].

In addition to binary assessment, the PQS demonstrates a spectrum of morphological appearances characterized by variations in fat stripe thickness, contour, and displacement relative to the distal radius [6,15]. Although classification systems have been proposed to describe these patterns, their clinical relevance and reproducibility remain insufficiently validated, thereby limiting the consistent application of the PQS in paediatric wrist trauma evaluation.

Thus, the present study aims to address this gap by determining (1) whether quantitative assessment of the PQS using standardized radiographic measurements can serve as a diagnostic adjunct for paediatric wrist fractures, and (2) whether specific PQS morphological patterns are associated with the presence or type of fracture.

## 2. Materials and Methods

### 2.1. Study Design and Population

This retrospective study was conducted at the University Hospital of Geneva and was approved by the Cantonal Commission of Ethics for Scientific Research of Geneva (CCER) in June 2015 before data collection (CER No. 15-119). It included paediatric patients who presented to the emergency department with wrist trauma between 1 January 2011 and 31 December 2014.

Inclusion criteria comprised patients presenting with wrist trauma, aged older than 3 years, who underwent radiographic evaluation within 72 h and had high-quality radiographs. High-quality radiographs were defined as images with adequate exposure, clear visualization of cortical margins, and appropriate positioning, including true AP and Lat views of the wrist, with the lateral projection demonstrating at least 50% overlap of the radius by the ulna. It should be noted that, in younger children, pain and limited cooperation may affect positioning and image quality, which may influence PQS evaluation and interpretation.

The exclusion criteria included patients who underwent alternative imaging modalities instead of dedicated wrist radiographs; fractures with displacement or angulation greater than 20°; diaphyseal fractures; and multiple fractures involving the radius or ulna. Additionally, patients with closed growth plate cartilage or radiographs deemed uninterpretable due to motion artifacts or poor image quality were excluded.

### 2.2. Radiographic Assessment

Standard AP and Lat X-ray views were obtained for all patients. Patients were then categorized into three groups based on their radiographic findings: (1) no fracture, (2) torus fracture, and (3) other non-displaced distal radius fractures. This group included metaphyseal distal radius fractures without cortical collapse (excluding torus fractures), suspected Salter–Harris type I–II physeal injuries without displacement, and incomplete fractures without angulation greater than 20°. Salter–Harris type I injuries were diagnosed based on clinical findings (e.g., localized physeal tenderness) as these fractures are often not directly visible on X-rays.

**Figure 1:**
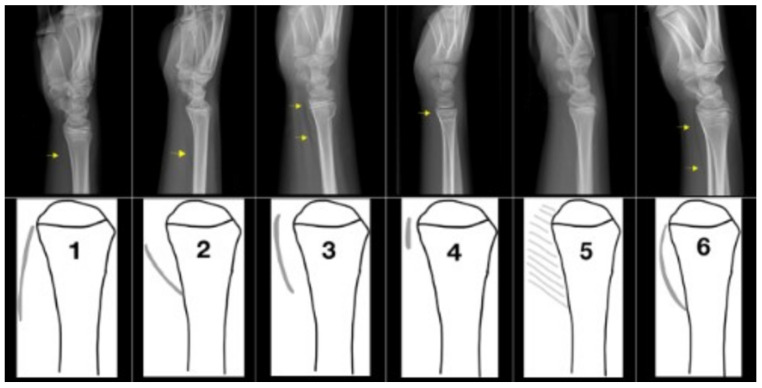
Illustration of the pronator quadratus sign (PQS) evaluation using a six-tier classification system. Representative lateral wrist radiographs (**upper row**) and corresponding schematic illustrations (**lower row**) demonstrate PQS types 1–6. Yellow arrows indicate the pronator quadratus shadow and the region used for PQS assessment.

Classification into these three diagnostic groups was performed by a senior Paediatric Orthopaedic Surgeon and an experienced Paediatric Radiologist. Isolated ulnar fractures were not analysed separately; in cases with associated ulnar involvement, classification was based on the distal radius fracture pattern. The PQS was evaluated using a six-tier morphological classification system describing the appearance of the pronator quadratus fat stripe based on predefined visual criteria reflecting its visibility, contour, and displacement relative to the distal radius. Type 1 corresponded to a normal appearance with a well-defined fat stripe and distal contact with the bone. Type 2 described a fat stripe with proximal bone contact. Type 3 represented a clearly elevated fat stripe without bone contact. Type 4 indicated an elevated straight fat stripe suggestive of soft tissue distortion. Type 5 was defined by the obliteration of the fat stripe, while Type 6 corresponded to a fat stripe with contact to the bone both proximally and distally. Representative examples of each PQS type are provided in Figure 1.

All radiographic analyses and measurements were performed using the HUG imaging software Weasis—Weasis Manager (version 3.8.2).

### 2.3. Primary Outcomes

**Figure 2:**
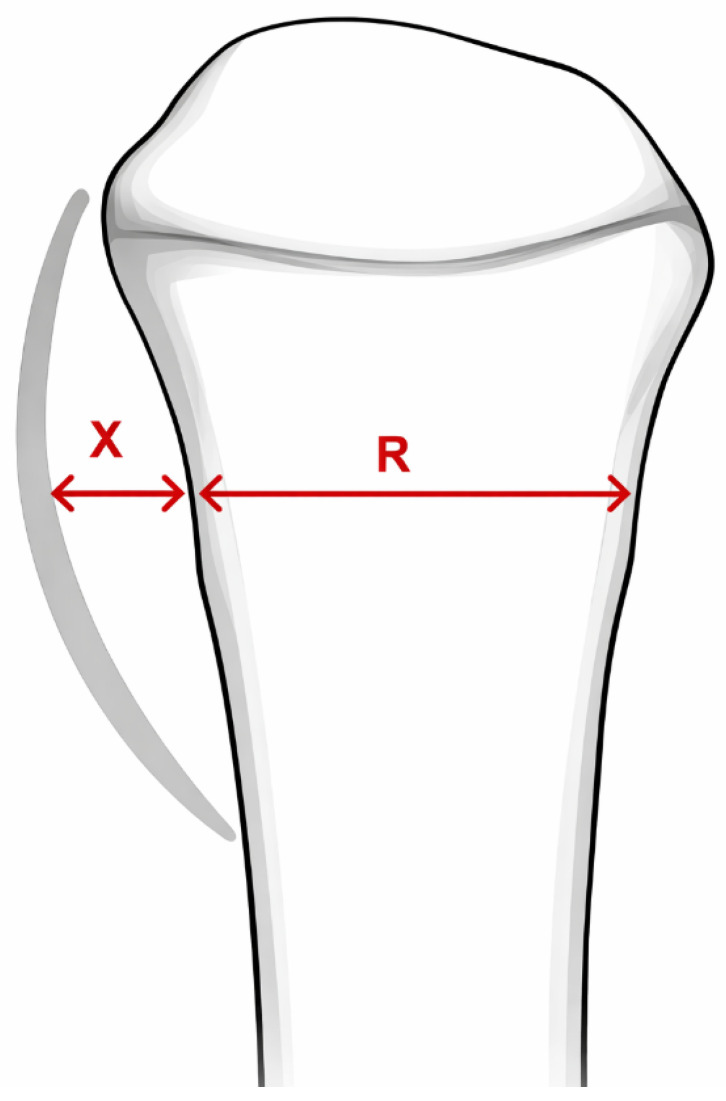
Illustration of the measurement principle of the PQS on a lateral radiograph. R represents the radial width measured at the reference level. X represents the distance (mm) from the anterior cortex from distal radius to the anterior layer of PQm, measured at the same level as R.

The X/R ratio was calculated to quantify PQS displacement relative to the radial width [18]. X was defined as the distance (mm) from the anterior cortex from distal radius to the anterior layer of PQm, while R represented the radial width (mm) at the same level. X and R were initially measured as distances and then expressed as a ratio to standardize PQS measurement across patients and avoid image calibration mismatches (Figure 2).

Quantitative X/R measurements were not possible in all radiographs because standardized lateral positioning and clear visualization of the pronator quadratus region were required for reliable measurement. Radiographs with suboptimal positioning, incomplete visualization, or insufficient image quality were excluded from the quantitative analysis.

### 2.4. Statistical Analysis

All statistical analyses were performed using R software (version 4.5.0, R Foundation for Statistical Computing, Vienna, Austria). Statistical significance was set at *p* < 0.05. The normality of the data distribution was evaluated using the Shapiro–Wilk test.

We assessed the diagnostic performance of the PQS for wrist fractures by analysing both the standardized X/R ratio (%) and PQS morphological types. The X/R ratio differences among no fracture, torus fracture, and other non-displaced distal radius fractures groups were evaluated using one-way ANOVA with Tukey post hoc tests, while its diagnostic performance was quantified through Receiver Operating Characteristic (ROC) curve analysis comparing no fracture versus torus/other non-displaced distal radius fractures separately using the *pROC* package and area under the curve (AUC) with Delong 95% confidence intervals were reported. Sensitivity and specificity were calculated at optimal thresholds determined using the Youden index.

Additionally, we examined associations between the six-tier PQS morphological classification and fracture types using Pearson’s chi-square tests with respective post hoc analysis.

### 2.5. Sample Size Considerations

Sample size calculations were performed in R software using the *pwr* package. Based on preliminary data showing an expected effect size (Cohen’s f = 0.30, α = 0.05, power = 0.80, and k = 3 groups) for X/R ratio differences between fracture groups, we calculated that 210 patients (70 per group) would provide 80% power at α = 0.05 using ANOVA. Our final sample of 247 patients (72 no fracture, 106 torus fracture, 69 non-displaced distal radius fractures) exceeded this requirement while accommodating potential exclusions.

## 3. Results

### 3.1. Population Characteristics

**Figure 3:**
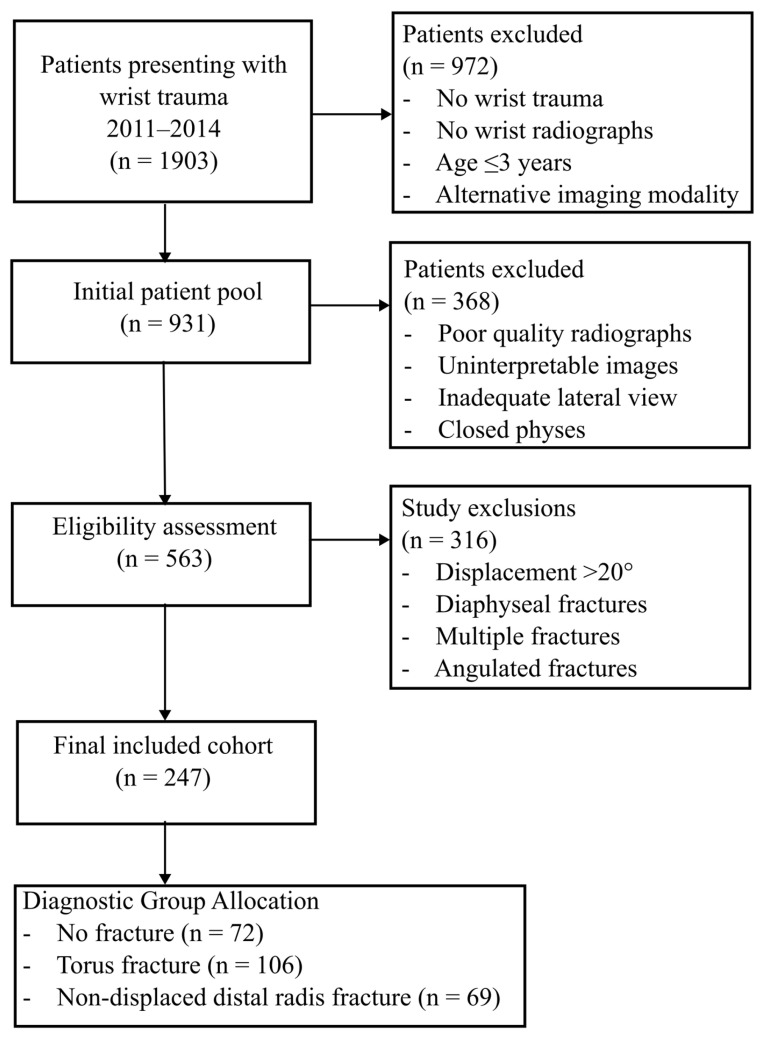
Flowchart illustrating patient identification, eligibility assessment, exclusions, and final diagnostic group allocation.

A total of 247 patients were included in the study. “No fracture” group consisted of 72 patients, “Torus fracture” group included 106 patients, and other non-displaced distal radius fractures group comprised 69 patients (Figure 3).

The proportion of female patients was 57% in no fracture group (*n* = 41), 45% in torus fracture group (*n* = 48), and 45% in non-displaced distal radius fractures group (*n* = 31), with no statistically significant difference between groups (*p* = 0.241). In contrast, the median age differed significantly: 11 years [IQR 9–13] in no fracture group, 10 years [IQR 8–12] in torus fracture group, and 11 years [IQR 7–12] in non-displaced distal radius fracture group (*p* = 0.005). Table 1 provides an overview of the demographic characteristics and fracture distribution among the study groups.

### 3.2. X/R Ratio Analysis

**Figure 4:**
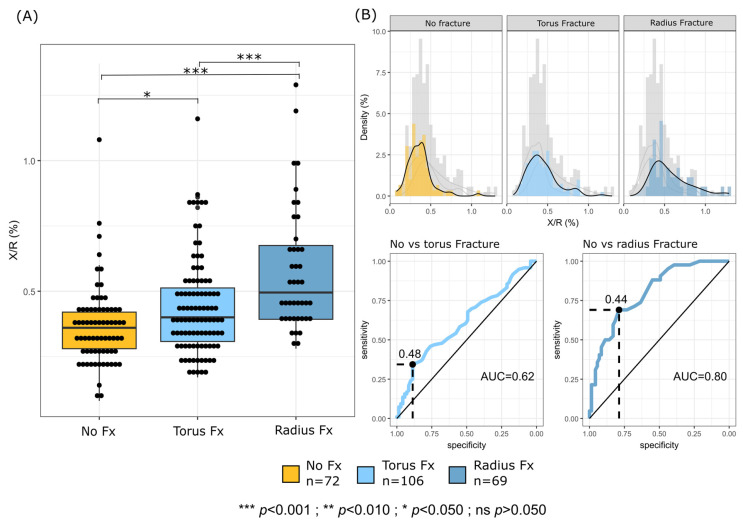
Predictive value of the PQS for wrist fractures by analysing the standardized X/R ratio (%). X was defined as the anterior distance (mm) from the distal radius to the external border of the PQm, while R represented the radial width (mm) at the same level. (**A**) Boxplots comparing X/R ratios among the no-fracture group (yellow, *n* = 72), torus fracture group (light blue, *n* = 106), and radius fracture group (dark blue, *n* = 69). Individual data points are shown as black dots. Horizontal brackets indicate significant differences between groups (*p* < 0.05, *** *p* < 0.001). (**B**) Upper panels show histograms and density plots of X/R ratios for each group; colored bars correspond to each study group, and gray bars represent the overall distribution. Lower panels show receiver operating characteristic (ROC) curves comparing the no-fracture group with the torus fracture group (left; AUC = 0.62) and with the radius fracture group (right; AUC = 0.80). Dashed lines indicate the optimal cutoff values (0.48 and 0.44, respectively).

The X/R ratio (%), quantifying the maximum anterior displacement of the PQS relative to the radial width, differed significantly across the three diagnostic groups (*p* < 0.001). The group without fracture had the lowest median X/R ratio at 0.36 [0.28–0.42], followed by torus fracture group at 0.40 [0.31–0.51], and non-displaced distal radius fractures group at 0.50 [0.39–0.68]. Post hoc analysis revealed statistically significant differences between all three groups (*p* < 0.001). These results indicate that higher X/R ratio values were associated with fracture presence and varied across diagnostic groups (Figure 4A).

ROC curve analysis demonstrated differential performance of the X/R ratio in fracture discrimination: while it showed limited ability to distinguish no fracture from torus fracture cases (AUC = 0.62, 95% CI 0.53–0.70; optimal cutoff = 0.48, sensitivity = 0.34, specificity = 0.89), it exhibited significantly better discrimination between no fracture and non-displaced distal radius fracture groups (AUC = 0.80, 95% CI 0.72–0.88; optimal cutoff = 0.44, sensitivity = 0.69, specificity = 0.79) (Figure 4B).

### 3.3. PQS Type & Subgroup Distribution

**Figure 5:**
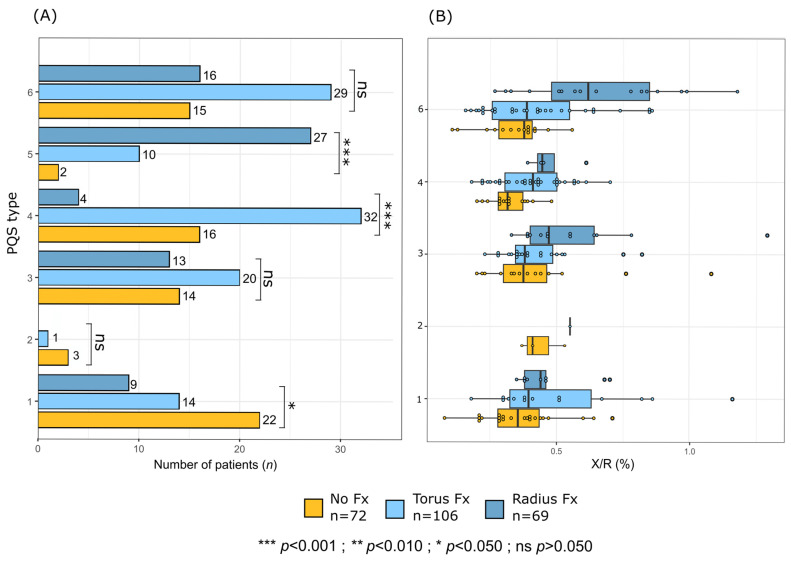
Associations between the six-tier pronator quadratus sign (PQS) classification and the fracture status. (**A**) Horizontal bar charts showing the number of patients in each PQS category (types 1–6) according to fracture status: no fracture (yellow, *n* = 72), torus fracture (light blue, *n* = 106), and radius fracture (dark blue, *n* = 69). Brackets indicate statistically significant differences among groups (*p* < 0.05, *** *p* < 0.001, ns = not significant). (**B**) Boxplots of the standardized X/R ratio (%) for each PQS category and fracture group. Circles represent individual patient measurements.

The distribution of PQS types differed significantly across the three groups (Figure 5A). PQS type 1 was identified in 31% of patients in no fracture group, compared to 13% in both torus and non-displaced distal radius fracture groups (*p* = 0.056). PQS type 4 was present in 22% of no fracture group, 30% of torus fracture group, and only 6% of non-displaced distal radius fracture group (*p* < 0.001). These findings suggest a strong association between PQS types 4 and 5 and the presence or specific type of fractures.

In contrast, no statistically significant differences were observed for PQS types 2 and 3 (*p* > 0.050).

As reported Figure 5B, the X/R ratio tends to be higher in the non-displaced distal radius fractures group compared to the no fracture and torus fracture groups for the PQS types 3 and 6.

## 4. Discussion

This study demonstrates that both quantitative (X/R ratio) and qualitative (PQS types) assessment of the PQS have diagnostic value in paediatric wrist trauma, with distinct clinical implications. Our results showed that the X/R ratio was significantly higher in patients with fractures, suggesting that PQS measurements may provide additional valuable diagnostic information. However, the modest AUC (0.62) for distinguishing no fracture from torus fractures suggests limited utility in borderline cases, possibly due to subtle soft tissue changes in torus injuries. The higher AUC (0.80) for non-displaced distal radius fractures supports the potential role of the X/R ratio as an adjunctive indicator rather than a standalone diagnostic tool for non-displaced distal radius fractures, particularly with a cutoff of 0.44 (sensitivity: 69%, specificity: 79%).

From a clinical perspective, the X/R ratio may assist in cases with equivocal radiographic findings by supporting decisions such as closer follow-up, temporary immobilization, or additional imaging. Nevertheless, given its overall moderate performance, it should be interpreted alongside clinical and radiographic findings rather than used in isolation.

While the overall PQS morphological classification alone showed limited predictive value, specific PQS types (notably types 4 and 5) demonstrated significant associations with fracture categories. Type 4’s prevalence in torus fractures (30%) and Type 5’s dominance in non-displaced distal radius fractures (39%) suggest these patterns may reflect differences in injury characteristics, although causal interpretation cannot be established. Notably, Type 1’s higher frequency in uninjured wrists (31% vs. 13%) could represent a normal variant, although its borderline significance (*p* = 0.056) warrants cautious interpretation. To our knowledge, few studies have examined fracture-specific PQS morphological patterns, with prior work largely relying on binary (present/absent) assessments.

Our findings both align with and diverge from those previously reported in the literature. These observations are also consistent with the recent systematic review and meta-analysis by Kadhmawi et al., which demonstrated moderate specificity but heterogeneous sensitivity of the PQS across studies and concluded that it should be interpreted as a supportive rather than definitive diagnostic feature [17]. In agreement with Fallahi et al. (2012), we found that disrupted and obliterated PQS patterns were more frequently associated with wrist fractures than raised patterns [15]. As Zammit-Maempel et al. (1988), we observed that PQS was absent in a substantial number of confirmed fracture cases, reflecting its limited sensitivity [11]. Similarly, Annamalai and Raby (2003) reported poor diagnostic performance of the PQS, which is consistent with our finding that PQS type alone did not reliably predict the fracture presence and its type [16].

On the other hand, our introduction of the X/R ratio offers a novel, quantitative measurement that may overcome some of the subjectivity and variability described by previous authors such as Sasaki and Sugioka (1989), who proposed a classification system without measurement standardization [5]. Importantly, the lack of standardized PQS assessment methods highlighted in the recent meta-analysis further supports the rationale for our quantitative X/R ratio approach in paediatric wrist trauma evaluation. These findings suggest that while visual categorization of PQS remains unreliable, standardized quantification of soft tissue displacement could enhance its clinical value as a diagnostic adjunct [17].

Several studies have evaluated the diagnostic value of the PQS, reporting varying degrees of sensitivity, specificity, and clinical reliability. MacEwan (1963) first described the PQS, observing alterations in the fat layer overlying the PQm in 295 of 300 patients with distal forearm fractures [10]. Curtis et al. (1984) later found a positive PQS in 80% of distal forearm fractures, supporting its role as a potentially useful early diagnostic marker [14]. In the same year, Zimmer (1984) emphasized the potential for both false-positive and false-negative PQS findings, attributing these to inflammatory conditions, suboptimal imaging, or timing immediately post-injury [13]. Zammit-Maempel et al. (1988) assessed over 1400 wrist injuries and found abnormal PQS in only 51% of confirmed fractures, highlighting its limited sensitivity [11]. Sasaki and Sugioka (1989) introduced a four-tier classification system for PQS morphology, suggesting that some PQS types may be more predictive of fracture presence than others [5]. However, Annamalai and Raby (2003) challenged the diagnostic accuracy of PQS, reporting a sensitivity of just 26% and specificity of 70% in detecting occult fractures [16]. Most recently, Fallahi et al. (2012) conducted a prospective MRI-validated study of 68 patients and found that while obliterated and disrupted PQS patterns were associated with fractures, the overall sensitivity and specificity were 65% and 69%, respectively, limiting its reliability as a standalone marker [15].

To address these limitations of conventional radiography, recent studies have explored the use of sonography to improve diagnostic performance in paediatric distal forearm injuries [19,20,21,22,23,24]. Similarly to the radiographic PQS, point-of-care ultrasound describes the “pronator quadratus hematoma” (PQH) sign, as reported by Snelling et al. (2022) [20], which reflects soft tissue swelling within the pronator quadratus muscle. In a prospective cohort of 38 paediatric patients, the PQH sign correctly identified all cortical breach fractures and non-fracture cases, with clear quantitative differences in muscle thickness between groups [20].

Taken together, these findings suggest that PQ-related changes—whether assessed by radiography or ultrasound—may represent early indicators of underlying injury and support a multimodal diagnostic approach integrating clinical assessment and complementary imaging modalities, particularly in paediatric populations where minimizing radiation exposure is an important consideration [20,23].

Several limitations should be considered when interpreting these findings. First, the retrospective design introduces potential selection bias, as only patients who underwent radiographic evaluation were included. Second, interobserver agreement was not formally assessed in this study. This is an important limitation because both PQS morphology classification and X/R ratio measurements may be influenced by observer interpretation, radiographic quality, and patient positioning. Third, the classification of PQS morphological types may have been influenced by interobserver variability, despite efforts to standardize interpretation. Technical and imaging-related factors may also have affected PQS assessment. Patient positioning and radiographic quality could influence PQS visibility and measurement accuracy, particularly in younger children. In addition, palmar (volar) cortical buckle fractures may directly alter the appearance of the pronator quadratus region, representing a potential confounding factor. Radiographs obtained in tertiary care centres may also be of higher quality than those acquired in smaller or resource-limited settings, which may affect the generalisability of PQS assessment. In addition, the reduced number of available X/R measurements, particularly in the non-displaced distal radius fracture group, may have introduced selection bias and influenced the reported diagnostic performance. Diagnostic and methodological limitations should also be acknowledged. The diagnosis of suspected Salter–Harris type I injuries was based on clinical findings rather than direct radiographic confirmation, which may have introduced diagnostic uncertainty and potential misclassification bias. Bilateral wrist radiographs were not routinely performed; therefore, intra-individual comparison of PQS between wrists was not assessed. Finally, the study did not incorporate an independent reference standard, such as MRI or CT, which limits the ability to determine whether PQS identifies truly occult fractures or simply correlates with radiographically visible injury patterns.

Future research should focus on validating the diagnostic performance of the X/R ratio through prospective studies with larger sample sizes and standardized imaging protocols. The integration of advanced imaging modalities, such as MRI or ultrasound, may provide further insight into occult fractures and associated soft tissue changes, particularly in settings where MRI is not routinely available. In addition, refinement of PQS classification criteria—potentially combining qualitative and quantitative approaches—may enhance its clinical applicability in paediatric wrist trauma assessment. Finally, evaluation of the cost-effectiveness of PQS-based assessment in resource-limited settings will be important to determine its broader clinical utility.

## 5. Conclusions

This study demonstrated that PQS measurements, particularly the X/R ratio, are significantly higher in patients with fractures, supporting its potential as a supplementary diagnostic tool. However, its morphological classification alone appears not consistently diagnostic of fractures. PQS, particularly the X/R ratio, shows promise as a supplementary diagnostic tool, but its independent diagnostic performance remains uncertain. Further studies are needed to establish its clinical utility and contribute to improving fracture assessment. Incorporating advanced imaging techniques and refining PQS classification systems may provide a more robust framework for utilizing PQS in clinical practice.

## Figures and Tables

**Table 1:** Population description of the three groups: (1) no fracture, (2) torus fracture, and (3) other non-displaced distal radius fractures.

	No Fracture (*n* = 72)	Torus Fracture (*n* = 106)	Non-Displaced Distal Radius Fractures (*n* = 69)	ANOVA	
				*p*-Value	Post Hoc
**Individual characteristics**					
Sex *n* (%)	F:41 (57%); M: 31 (43%)	F:48 (45%); M: 58 (55%):	F:31 (45%); M: 38 (55%)	0.241	-
Age (years)	11 [9:13]	10 [8:12]	11 [7:12]	0.001 ***	a, b
**PQS type *n* (%)**					
PQS 1	22 (31%)	14 (13%)	9 (13%)	0.056	-
PQS 2	3 (6%)	1 (0%)	0 (0%)	0.317	-
PQS 3	14 (19%)	20 (19%)	13 (19%)	0.401	-
PQS 4	16 (22%)	32 (30%)	4 (6%)	<0.001 ***	a, c
PQS 5	2 (3%)	10 (9%)	27 (39%)	<0.001 ***	b, c
PQS 6	15 (21%)	29 (27%)	16 (23%)	0.047 *	-
**Radiological** **parameters**	***n* = 71**	***n* = 88**	***n* = 42**		
X/R (%R)	0.36 [0.28; 0.42]	0.40 [0.31; 0.51]	0.50 [0.39; 0.68]	<0.001 ***	a, b, c

Results are presented as median [IQR = interquartile range] or *n* (%). Min is minimum and max is maximum. Level of significance was reported as follows: ***: *p* < 0.001, **: *p* < 0.01; *: *p* < 0.05. Tukey post hoc tests significance (*p* < 0.05) was reported as: a: No fracture vs torus fracture, b: no fracture vs non-displaced distal radius fractures; c: torus fracture vs non-displaced distal radius fractures.

## Data Availability

The data supporting the findings of this study are not openly available due to sensitivity concerns. They are accessible from the corresponding author upon reasonable request. The data are stored in a controlled-access repository at Geneva University Hospitals, Switzerland.

## Abbreviations

The following abbreviations are used in this manuscript:

PQS	Pronator Quadratus Sign
PQFP	Pronator Quadratus fat pad sign
AP	Anteroposterior
Lat	Lateral
PQm	Pronator Quadratus Muscle
MRI	Magnetic Resonance Imaging
CCER	Cantonal Commission of Ethics for Research
HUG	Geneva University Hospitals
IQR	Interquartile Range
ANOVA	Analysis of Variance
ROC	Receiver Operating Characteristic
AUC	Area Under the Curve
CI	Confidence Interval
